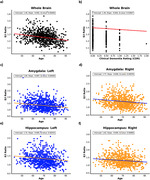# Altered Regional and Whole‐Brain Excitation/Inhibition Balance Correlates with Alzheimer's Disease Risk Factors

**DOI:** 10.1002/alz.092452

**Published:** 2025-01-09

**Authors:** Andrew P Burns, Igor Fortel, Barbara B. Bendlin, Liang Zhan, Alex Leow

**Affiliations:** ^1^ University of Illinois at Chicago, Chicago, IL USA; ^2^ University of Wisconsin ‐ Madison, Madison, WI USA; ^3^ University of Chicago, Chicago, IL USA; ^4^ Department of Medicine, Geriatrics Division, University of Wisconsin School of Medicine and Public Health, Madison, WI USA; ^5^ University of Pittsburgh, Pittsburgh, PA USA

## Abstract

**Background:**

Research indicates that the brain operates near a critical excitation/inhibition (E/I) balance point, which, when disrupted, correlates with Alzheimer’s Disease (AD) risk factors such as APOE genotype and sex. Utilizing our established multimodal imaging method (Fortel, Igor, et al. Network Neuroscience 6.2 (2022): 420‐444.), we investigated the E/I balance in cognitively normal and impaired individuals, focusing on factors like APOE status, amyloid status, age, sex, and dementia ratings.

**Method:**

We analyzed 593 participants, aged 43‐92 (m = 62), including 495 cognitively intact and 98 impaired individuals, through 871 E/I ratio assessments (207 had multiple assessments). Participants underwent T1‐weighted, resting‐state, and diffusion‐weighted MRI to construct hybrid resting state structural connectomes for 105 regions using a recent multimodal connectome technique to detect whole‐brain and region‐specific E/I ratios. We employed two linear mixed models to correlate E/I ratios with age, sex, e4+/e4‐ status (binary) or APOE‐npscore (continuous), whole‐brain amyloid status (binary), and clinical dementia rating, adjusting for multiple comparisons. Patient IDs were used as random effects in the models to account for repeated measurements, with no interaction effects included. All imaging and variables were obtained from OASIS‐3: Longitudinal Multimodal Neuroimaging: Principal Investigators: T. Benzinger, D. Marcus, J. Morris; NIH P30 AG066444, P50 AG00561, P30 NS09857781, P01 AG026276, P01 AG003991, R01 AG043434, UL1 TR000448, R01 EB009352. AV‐45 doses were provided by Avid Radiopharmaceuticals, a wholly owned subsidiary of Eli Lilly.

**Result:**

Both linear mixed models identified 26, significant E/I variations across the brain related to age, clinical dementia rating, and sex. Specific AD‐relevant regions such as the hippocampus and amygdala (Figure 1) show notable E/I changes. Whole brain E/I was negatively correlated with the APOE‐ε4 allele in the binary APOE status model but not in the continuous APOE‐npscore model.

**Conclusion:**

We demonstrated significant E/I ratio alterations in the whole brain and known AD relevant regions, such as the hippocampus and amygdala, against the largest risk factor of AD (age), clinical dementia rating, and binary APOE status. Our findings are consistent with past studies exhibiting correlations between altered E/I metrics and known AD risk factors.